# Use of physical restraints among patients with bipolar disorder in Ethiopian Mental Specialized Hospital, outpatient department: cross-sectional study

**DOI:** 10.1186/s40345-017-0084-6

**Published:** 2017-03-23

**Authors:** Habte Belete

**Affiliations:** Psychiatry Department, College of Medicine and Health Science, Bahir Dar University, Bahir Dar, Ethiopia

**Keywords:** Restraint, Bipolar disorder, Ethiopia

## Abstract

**Background:**

Even though United Nation announced that all persons with a mental illness shall be treated with humanity and respect for the inherent dignity of the human being, up to now, the use of coercion (physical restrain) is still considered as unavoidable in managing abnormal behavior of psychiatric patients. But, there is no information regarding the magnitude and contributing factors of physical restrain among bipolar patients in low-income countries like Ethiopia.

**Methods:**

A cross-sectional study was conducted at Amanuel Mental Specialized Hospital from May 1 to June 1, 2015 among 400 participants who were selected by systematic random sampling technique. Data were collected by interviewing; adjusted odd ratios (AOR) with 95% confidence intervals (CI) were used and *p* value <0.05 was considered as statistically significant.

**Results:**

The prevalence of physical restrain was 65%. Factors like, having two or more episodes [AOR = 1.84 95% CI (1.16, 2.93)], history of aggression [AOR = 2.14, 95% CI (1.26, 3.63)], comorbid illness [AOR = 1.76, 95% CI (1.26, 3.63)], use of antipsychotic [AOR = 1.79, 95% CI (1.08, 2.95)] and current use of Khat [AOR = 1.83, 95% CI (1.10, 3.04)] were associated significantly.

**Conclusions:**

The prevalence of physical restraint is found high among bipolar patients and it needs public health attention.

## Background

Mechanical restraint is an intervention currently used in mental health services and other related settings to control or manage abnormal behavior.

Restraint includes use of bodily force (physical restraint) or a device (mechanical restraint) to control a person’s freedom of movement. It also refers to the use of medication (chemical restraint) to control a person’s behavior (McSherry 2013). Even though United Nation announced that all persons with a mental illness shall be treated with humanity and respect for the inherent dignity of the human person (United Nations General Assembly 2008); up to now, the use of coercion is occasionally considered as unavoidable in managing aggressive behavior. But seclusion and physical restraint were sometimes used more than necessary (McCann et al. 2014).

Studies in western countries elucidate that the use of coercive measurement of mental health care is quite different in terms of magnitude, ethical acceptability, effectiveness and duration of management among countries (Steinert et al. 2010; Mayoral and Torres 2005; Sailas and Wahlbeck 2005) and quality of psychiatric service also affected by the use of coercive management (Kallert et al. 2005). Patients also express their felling as being treated involuntarily for psychiatric care is a balancing between good opportunity and great loss (Johansson and Lundman 2002) and psychiatric patients who were suffer to coercive measurement were less satisfied with the psychiatric service compared with none coercively treated patients (Katsakou et al. 2010).

Many restrained psychiatric patients had developed muscular atrophy due to their restraint and all required physical therapies during their hospitalization (Puteh et al. 2011). Even though seclusion and restraint practice are used to control dangerous behavior (Needham et al. 2004), an Italian study showed that absence of seclusion and restraint practice resulted low violence in psychiatric wards (Raja and Azzoni 2005). The reasons for restraint are multiple and studies blamed for aggressive behavior of the individuals (Raboch et al. 2010).

A community study in Indonesia indicates that psychiatric patients who have lived in rural district had restrained up to 21 years (Minas and Diatri 2008). A qualitative study in Australia explored that seclusion and restraint practices were considered as unnecessarily overused and exacerbating problems for individuals, careers and staffs in mental health settings (Brophy et al. 2016).

The prevalence of physical restraints varies from different settings and cultures through the world. In specific and large-scale population based, cross-sectional studies indicate that prevalence of physical restraints varies from 10% in Finland (Kaltiala-Heino et al. 2000) to 78% in Switzerland (Needham et al. 2002). A large prospective study among ten western countries shows that incidence of restraint was ranged from 17% in Sweden to 69% in Greece (Raboch et al. 2010). The prevalence of coercive management is varying within settings. A multi-center study in Norway showed that prevalence of coercive management varies from 0 to 88% across wards and among involuntary admitted patients 10% of them were restrained (Husum et al. 2010). From Finland among hospitalized patients, coercion and restrictions were applied to 32% of the patients while mechanical restraints were used on 10% of the patients (Kaltiala-Heino et al. 2000).

In Canadian intensive care unit, the prevalence of physical restraint was 53% and predictors were use of sedative, analgesic, and antipsychotic drugs, agitation, and occurrence of an adverse event (Luk et al. 2014). In an Italian retrospective study, the main causes of mechanical restraint among psychiatric patients were to manage aggressive behavior of male patients, the presence of organic comorbidity and neurocognitive disorders (Di Lorenzo et al. 2014).

The qualitative study in Iran explored that physical restraint is extensively used as one of the main strategies to control psychiatric patients, despite having negative consequences (Moghadam et al. 2014) and also other case–control finding in the psychiatric emergency wards states that patients with aggressive behavior, methamphetamine-induced psychotic disorder and bipolar I disorder in manic episode were more suffering restrained during their hospitalization (Hadi et al. 2015).

In an Indian survey study, among psychiatrists (278), 80% of them sometimes used physical restraints as the management of their mental ill patients, but only 70% of them took informed consent from the relatives before they order physical restrain for their patients (Khastgir et al. 2003).

Despite this higher prevalence of physical restraints among psychiatric patients, there are no published data onto Ethiopia, and also in Sub-Saharan regions regarding physical restraints among bipolar patients. Therefore, this study was intended to assess the prevalence of physical restraints and predictors among bipolar patients those who were presenting at Amanual Mental Specialized Hospital, Addis Ababa, Ethiopia.

## Methods

### Study setting

Hospital-based cross-sectional survey was implemented among bipolar patients at Amanuel Mental Specialized Hospital outpatient department. Amanuel Mental Specialized Hospital is the only mental hospital in the country, which host many referral cases throughout the country and has 15 outpatient departments, 8 of them serve for more than 11,500 bipolar follow-up patients per year. Amanuel Mental Specialized Hospital has 300 beds which serve for adult psychiatry inpatients, emergency, forensic and addiction service.

### Participants

Patients clinically diagnosed (with Diagnostic and statistical manual of mental disorders, 5th edition) for bipolar disorder (any type of bipolar disorder) by psychiatrists and mental health professional specialists (master, degree holder on mental health) at Amanuel Mental Specialized Hospital were taken as source population. The source of population was taken as those patients who visit regularly the hospital in 1 year, whose count was 11,500 bipolar patients that visit per year averagely.

Study population was considered for those patients who were available at outpatient departments on the time of data collection and fulfill the inclusion criteria. Bipolar patients, whose age 18 years and above, were included in the study; but those who were seriously ill (patients who emotionally disturbed and unable to maintain normal conversation) and unable to communicate were not included.

Among 423 potential participants 400 had complete the interview; but 10 denied participating; 8 failed to complete the interview and 5 were excluded. Systematic random sampling technique was used to select 423 participants among 954 bipolar patients who visit the hospital per month averagely and the sampling fraction was two. Data were collected by well-trained (for the questionnaire), degree-holding psychiatric nurses with interviewing patients using questionnaire which is translated to a local language (Amharic, national working language of Ethiopia), from May 1 to June 1, 2015.

### Instrument

In this survey study, physical restraint was assessed by asking the patients, relatives and reviewing their medical records for the experience management of physical restraints since their morbidity. The site of restraints included all type of physical restraints like hands, legs or both.

Socioeconomic status of the study participants has been assessed using principal component analysis in which Eigen values greater than one were used as an extraction and factors to be extracted was fixed to five (from the lowest to highest; since Ethiopian demographic and health survey system use it). Their clinical variables were assessed by the standardized tools such as drug adherence was assessed using Morisky Medication Adherence Scale 8 item tool (Morisky et al. 2008); social support assessed using Oslo-3 Social support Scale (Bøen 2012); perceived stress assessed by perceived stress scale 10 item tool (Cohen et al. 1983); and current substance use was assessed by adopted alcohol smoking and substance involvement screening test (Humeniuk et al. 2008).

### Analysis

The collected data were entered into Epi Data 3.1 and analyzed using Statistical Package for Social Science version 20. The respondents’ descriptive statistic was shown and multivariate and binary logistic regression analysis was used to select predictors that associated with physical restraint. Association was presented by odds ratios by taking 95% confidence intervals (CI) and *p* values less than 0.05 were taken as statistically significant.

### Ethical clearance

Ethical clearance taken from Ethical Review Board of University of Gondar and Amanuel Mental Specialized Hospital as well as formal permission letters were taken from administrative staff of the hospital. Written consent taken from the participants and confidentiality were kept throughout the process by unanimous questionnaire and omitting personal identification.

## Results

The total participants were 400 bipolar patients in the study and 229 (57.2%) were females. The median age of participants was 32 years with inter quartile range of 14 and most of the participants 294 (73.5%) was from urban (Table 1).

**Table 1:** Socio-demographic factors of the participants at Amanuel Mental Specialized Hospital, 2015

Characteristics	Frequency	Percent (%)
Age		
18–26	104	26.0
27–32	106	26.5
33–40	100	25.0
41–75	90	22.5
Residency		
Urban	294	73.5
Rural	106	26.5
Religion		
Orthodox	239	59.7
Muslim	94	23.5
Protestant	61	15.3
Catholic	6	1.5
Marital status		
Single	189	47.2
Married	174	43.5
Divorced/widowed/separated	37	9.3
Ethnicity		
Amahara	139	34.7
Oromo	132	33.0
Gurage	79	19.7
Tigray	23	5.8
Others^a^	27	6.8
Occupation		
Government employee	47	11.8
Non government employee	35	8.8
Farmer	34	8.5
Merchant	59	14.7
Student	26	6.5
Housewife	60	15.0
Daily worker	20	5.0
Jobless	119	29.7
Living condition		
With family	349	87.2
Alone	51	12.8
Wealth index		
Lowest	82	20.5
Second	88	22.0
Middle	77	19.2
Fourth	84	21.0
Highest	69	17.3

^a^Others (Wolaita, Sidama, Gamo)

### Clinical factors

In relation to clinical factors, among the total participants (400); 140 (35%) of them had poor adherence to their medication while 173 (43.2%) of them had psychotic symptoms, 192 (48%) of had depression symptoms and 101 (25.2%) of them had manic symptoms during the survey (Table 2).

**Table 2:** Participants and factors related to physical restraints at Amanuel Mental Specialized Hospital, 2015

Variable	Frequency	Percentage (%)
History of aggression		
Yes	323	80.8
No	77	19.2
Number of episode		
First episode	115	28.8
Second episode	128	32.0
Third episode	53	13.2
Fourth episode	22	5.5
More than four episode	82	20.5
Type of current medication		
Mood stabilizer	238	59.5
Antidepressant	57	14.5
Antipsychotic	269	65.5
Antipsychotic and mood stabilizer (combination)	181	44.0
Drug adherence		
Poor adherence	140	35
Moderate adherence	129	32.2
High adherence	131	32.8
Perceived stress		
Low perceived stress	116	29.0
Average perceived stress	78	19.5
High perceived stress	206	51.5
Social support		
Poor social support	138	34.5
Moderate social support	178	44.5
Strong social support	84	21.0
Duration of morbidity		
Less than 1 year	20	5
1–5 years	168	42
More than 5 years	212	53
Current use of substance		
Alcohol	108	27.3
Khat	123	30.8
Smoking	74	18.5

### Physical restraints

From the total 400 participants, 260 (65.0%) had managed with physical restraints for their abnormal behavior and regarding the setting of restrained, 188 (47%) of the participants was restrained at home while 22 (5.5%) was restrained at hospital, but 50 (12.5%) was restrained in both hospital and in their home. All restrained patients chained either their hand or legs’ or both. Regarding their time of restraint, 215 (53.8%) of the respondents had restrained during day time, 38 (9.5%) during night, and the remaining during both time.

Among 260 physically restrained patients, 75.8% of them had more than one episode of bipolar disorder; 56 (21.5%) of them had more than four episodes and 223 (85.8%) of them had history of aggressive behavior. Regarding comorbid illness of the total participants, 133 (33.3%) had comorbid illness [schizophrenia (27), epilepsy (23), substance-related problems (30), mental retardation (25), brain injury 28)] and among these 98 (37.7%) had managed with physical restraints. Since this mental hospital is the only mental hospital of the country that hosts many relapsed and chronic patients from every side of the country; the comorbidity of schizophrenia and other mentioned disorders looks unusual. The magnitude of current substance usage among physical restrained patients was high and 77 (29.6%) use alcohol; 54 (20.8%) smoke tobacco and 95 (36.5%) use Khat.

### Bivariate and multivariate analysis

All variables were processed on bivariate analysis and only six variables (sex, past history of aggression, use of antipsychotic drugs, comorbid illness, more than one episode of bipolar disorder, and current use of Khat) were found to be significant and consider for multivariate regression. Finally, after bivariate and multivariate regression analysis of physical restraint in relation to all independent variables; history of past aggression, use of antipsychotic drugs, comorbid illness, more than one episode of bipolar disorder, and current use of Khat were found to be statistically significant (Table 3).

**Table 3:** Factors for physical restraints on bipolar patients at Amanuel Mental Specialized Hospital, 2015

Explanatory variables	Physical restrained		Crude odd ratio (95% CI)	Adjusted odd ratio (AOR) 95% CI
	Yes	No		
Sex				
Male	122	49	1.64 (1.07, 2.51)*	1.46 (0.93, 2.23)
Female	138	91	1.00	1.00
Number of episode				
One episode	63	52	1.00	1.00
More than one episode	197	88	1.85 (1.18, 2.88)*	1.84 (1.16, 2.93)**
History of aggression				
Yes	223	100	2.41 (1.45, 3.99)*	2.14 (1.26, 3.63)**
No	37	40	1.00	1.00
Comorbid illness				
Yes	98	35	1.81 (1.15, 2.87)*	1.76 (1.09, 2.84)
No	162	105	1.00	1.00
Current use of Khat				
Yes	95	28	2.30 (1.42, 3.74)*	1.83 (1.10, 3.04)**
No	165	112	1.00	1.00
Use of antipsychotic				
Yes	91	30	1.97 (1.13, 3.18)*	1.79 (1.08, 2.95)**
No	169	110	1.00	1.00

* (*p* < 0.05) and ** (significantly associated), 1.00 (reference)

## Discussion

Physical restraint is an intervention mostly used in mental health services and other related settings to control or manage abnormal behavior. The management modality with physical restraints resulted patients with various physical injuries and psychological distress.

The prevalence of physical restraint is higher in this study when compared to the studies done in various countries like 10% in Finland (Kaltiala-Heino et al. 2000), 17% in Sweden (Raboch et al. 2010), lower than 78% in Switzerland (Needham et al. 2002) and 69% in Greece (Raboch et al. 2010). The factors like setting and methodological differences, clinical and socio-cultural factors might be responsible for this inconsistency.

After logistic regression analysis, those participants who had two or more episodes had an increase restrain by almost two times than had only one episode [AOR = 1.84 95% CI (1.16, 2.93)], which may be due to recurrence of the illness whereby increasing the chance of managed with restrained; but this finding was contradicted by a study in Iran in which those patients admitted for the first time were more frequently restrained than who had history of past hospitalization (Hadi et al. 2015).

Those patients who had history of aggression were two times more likely to managed with restrained as compared with those do not have aggressive behavior with odds of [AOR = 2.14, 95% CI (1.26, 3.63)], which coincide with Indonesia (Puteh et al. 2011) and Iran (Hadi et al. 2015). This may be due to the relation between being aggressive and the management by physical restrained for this behavior in the community and mental health care setting.

The use of Khat among bipolar patients was almost two times more likely managed with physical restrained than patients who had not used Khat [AOR = 1.83, 95% CI (1.10, 3.04)]. This may be related to use of substances increases the impulsivity, aggressiveness and emotionality of bipolar patients (Webb et al. 2014; Garno et al. 2008). The possible justification may be that use of Khat (since it is a stimulant) had influence on the brain and worsening of active symptoms.

Those patients who had comorbid illness were almost two times more likely to be managed with physical restrained as compared with those do not have comorbid illness with odds of [AOR = 1.76, 95% CI (1.26, 3.63)], which is in line with a study in Italy (Di Lorenzo et al. 2014). This may be due to the relation between having additional neurological and mental illness which leads patients to be more aggressive and physical restraint may be used to control for this behavior.

Regarding to their medication, patients who had use antipsychotic had almost two times more likely restrained than patients who had not used [AOR = 1.79, 95% CI (1.08, 2.95)], which coincides with Canadian study (Luk et al. 2014). The possible explanation for this may be that patients who had psychotic symptoms may have aggressive behavior and leads them to be restrained.

## Conclusions

The use of physical restraint is high among Ethiopian bipolar patients and needed public health attention as well ethical concerns. Numbers of episodes, history of past aggression, comorbid illness, use of antipsychotic and current use of Khat were found to be significantly associated with physical restraint.

## Limitation

The study was designed as a cross-sectional one that cannot show the temporal cause–effect association with factors and physical restraint. In this study, types of bipolar disorders were not assessing separately whether it has association or not with physical restrained. Current use of substance may not affect the lifetime use of coercive measurements.

## Abbreviations

AOR: adjusted odd ratio
CI: confidence interval
USA: United State of America
